# Facile Fabrication of MnO_2_/Graphene/Ni Foam Composites for High-Performance Supercapacitors

**DOI:** 10.3390/nano11102736

**Published:** 2021-10-15

**Authors:** Rui Liu, Rui Jiang, Yu-Han Chu, Wein-Duo Yang

**Affiliations:** 1 Center of Pharmaceutical Engineering and Technology, School of Pharmacy, Harbin University of Commerce, Harbin 150076, China; liur@hrbcu.edu.cn (R.L.); liurui19992007@163.com (R.J.); 2 Department of Chemical and Materials Engineering, National Kaohsiung University of Science and Technology, Kaohsiung 80778, Taiwan; churester@gmail.com

**Keywords:** MnO_2_, graphene, buffer layer, supercapacitors

## Abstract

A novel MnO_2_/graphene/Ni foam electrode was fabricated via the impregnation and electrochemical deposition technique with Ni foams serving as substrates and graphene serving as a buffer layer for the enhanced conductivity of MnO_2_. The samples were characterized using X-ray diffraction (XRD), Raman spectroscopy, scanning electron microscopy (SEM), and X-ray photoelectron spectroscopy (XPS). Compared with other methods, our strategy avoids using surfactants and high-temperature treatments. The electrodes exhibited excellent electrochemical performance, high capabilities, and a long cycle life. Various electrochemical properties were systematically studied using cyclic voltammetry and electrochemical impedance spectroscopy. The results showed that the specific capacitance of the MnO_2_/graphene/Ni composite prepared at 1 mA cm^−2^ of electrodeposition could achieve a scan rate of 10 mV s^−1^ at 292.8 F g^−1^, which confirmed that the graphene layer could remarkably improve electron transfer at the electrolyte–electrode interface. The capacitance retention was about 90% after 5000 cycles. Additionally, a MnO_2_/graphene//graphene asymmetric supercapacitor was assembled and it exhibited a high-energy density of 91 Wh kg^−1^ as well as had an excellent power density of 400 W kg^−1^ at 1 A g^−1^. It is speculated that the strong adhesion between the graphene and MnO_2_ can provide a compact structure to enhance the mechanical stability, which can be applied as a new method for energy storage devices.

## 1. Introduction

Supercapacitors are widely used in electric vehicles, electronic memory components, transportation facilities, computers and other communication equipment, and household appliances, which have attracted a great deal of attention and occupy a crucial position in energy-storage devices [1]. Supercapacitors have many advantages, such as their fast charge–discharge rate, low cost, excellent cycle life, and high-power density [2,3]. However, their low-energy density and conductivity have emerged as two obstacles limiting further practical application. Researchers have carried out a series of research projects on the matter of improvements to the energy storage density and cycle life. Among the various metal oxides, manganese dioxide (MnO_2_) has the advantages of having a low cost, environmental benignity, a high stability, and flexibility, thus it is often used as an electrode for supercapacitors [4,5].

The key to achieving a high-energy density and long cycle life is to fabricate novel electrode materials containing a design morphology, structure, and appropriate electrolytes. However, a major drawback for MnO_2_ as a supercapacitor material is its poor conductivity, which severely limits its applications. The above-mentioned deficiency can be improved by introducing active materials, such as carbon materials [6,7], conductive polymers [8,9], or transition metal oxides [10]. In recent years, graphene, a nano-carbon structure, has attracted a great deal of attention due to its excellent electronic and mechanical properties, chemical stability, and high specific surface area [11,12]. Therefore, some researchers have reported that MnO_2_/graphene composites can be used as a supercapacitor. Li et al. [13] fabricated a three-dimensional MnO_2_/reduced graphene oxide composite, in which the composite displayed a high specific capacitance and excellent capacitance retention after 1000 cycles. Yu et al. [14] developed a novel method to greatly improve the supercapacitor performance of graphene/MnO_2_-based nano-structured electrodes. Moreover, the composite exhibited excellent cycling performance in capacitance retention (>95%) over 3000 cycles. Sun et al. [15] constructed free standing 3D graphite and MnO_2_ nanoflake composites through a hydrothermal reaction. The 3D graphite network offers a pathway with a fast electron transport, while the MnO_2_ nanoflakes emanate from the surface backbone to minimize the interfacial contact resistance. Generally, electrochemical electrodes have also suffered from inadequate contact due to their low surface areas [16]. Numerous efforts have been made to improve these defective electrodes [17,18,19,20]. Huang et al. [20] reported a simple and convenient method to prepare supercapacitor electrodes using microwave-assisted synthesis, where graphene was deposited on Ni foam with a large-area substrate. 

In addition, from the perspective of the fabrication process, the fragile structure and weak contact between graphene and MnO_2_ result in poor conductivity and weak mechanical properties, which inhibits the electrochemical properties and stability of the capacitors. Several methods have been reported for the fabrication of MnO_2_/graphene/Ni foams, such as the hydrothermal method [14] and chemical bath deposition [20]. The rGO-MnO_2_ synthesized using the hydrothermal method experiences severe agglomeration, which decreases the conductivity and surface area of the electrode. Among these methods, collapses and cracks are serious problems during the preparation process; therefore, it is detrimental to the electrochemical performance and cycle life of capacitors. Firstly, graphene was completely adsorbed into the Ni foams using ultrasonic impregnation, with a large and close contact with the Ni foam substrate with a high mechanical strength. In using this method, the required thickness of film can be coated on the substrate, which is a simple process. Then, MnO_2_ is coated on the surface of the graphene/Ni foams using an electrochemical deposition method, with improvements to the speed of the electron transfers. Furthermore, the working electrode made using this technology has a high surface area and higher diffusion rate of the electrolytes.

In recent years, the electrochemical exfoliation method has been used to prepare graphene. Using the chemical effect of electric currents, the graphite electrode of the anode is exfoliated, forming graphene fragments in the electrolyte. Then, filtration and centrifugal purification are used to transfer the as-prepared graphene to the desired solution. The electrochemical stripping method has the advantages of simple operation, low cost, fast reaction time, and environmental friendliness. Khaled et al. [21] studied the influence of graphene obtained from electrolytic graphite with various inorganic salt solutions as electrolytes. They found that electrolyte containing SO_4_^2−^ exhibited a significant exfoliation efficiency. Compared with other anions, regarding the exfoliation efficiency of SO_4_^2−^, it is inferred that the reduction potential of SO_4_^2−^ is lower (+0.20 V), that is, it is easier to generate SO_2_ gas and sulfate ions can effectively exfoliate graphite [22].

In the present work, we report a novel route to produce graphene and MnO_2_ anchored onto Ni foam substrates. Graphene obtained using SO_4_^2−^ to exfoliate graphite is very environmentally friendly. The graphene was coated onto the Ni foams using the impregnation method and then the nanostructured porous MnO_2_ nanoparticles were precipitated onto the skeleton of the graphene/Ni composite foam via electrochemical deposition at a constant current. Compared with other methods, our strategy avoids using surfactants or high-temperature treatments. This structural design will enhance the transport rate of electrons and electrolyte ions during the electrode reaction. The MnO_2_/graphene/Ni composite greatly improves capacitor performance, with a specific capacity of 292.8 F g^−1^, which is about four times higher than that of graphene/Ni, and about five times higher than that of MnO_2_/Ni. It is expected that the MnO_2_/graphene/Ni electrode can obtain a higher specific capacitance and outstanding capacitance stability. This work offers new insights to consider in the construction of composite electrodes for high-performance supercapacitors. 

## 2. Materials and Methods

### 2.1. Materials

All chemicals used in this work were of analytical grade and were used without further purification. Nickel foam (98% purity, Fluka), manganese acetate tetrahydrate (99.9%), dimethylsulfoxide (99.9%), sodium sulfate (98%), ethanol (99%), acetone (99%), and ammonium acetate (99.9%) were from Sigma Aldrich. Sodium sulfate (≥98% purity, Honeywell) was used as the electrolyte.

### 2.2. Fabrication of Graphene/Ni Foam Electrodes

The anodic stripping method was used to synthesize graphene. A total of 0.1 M of ammonium sulfate was employed as the electrolyte, in which the SO_4_^2−^ was intercalated between the graphite layers, and the stripped graphene sheets floated in the electrolyte. Finally, the graphene was uniformly dispersed in the electrolyte using ultrasonic waves. The reaction time was 2 h. The electrolysis cell voltage was fixed at 15 V. After electrolysis, the electrolyte was filtered by vacuum suction and then the-obtained graphene was dried in a freeze-dryer. Finally, the obtained graphene powder was dispersed in 95% alcohol and shaken for 1 h. The concentration of the prepared graphene dispersion was approximately 1.0 mg mL^−1^.

Before the graphene immersion process, the Ni foam was polished mechanically and cleaned in acetone, nitric acid, and deionized (DI) water using ultrasonication for 10 min. Then, the blank nickel mesh was immersed in the graphene solution for 10 min. The electrode sheet was placed in an oven at 100 °C for 1 h to adhere the graphene onto the nickel foam.

### 2.3. Synthesis of MnO_2_/Graphene/Ni Composites

An electrochemical deposition technique was used for the three-electrode chemical reaction (reference electrode: Ag/AgCl; working electrode: graphene/Ni foam; counter electrode: Pt). The electrolyte contained 0.1 M Mn(CH_3_COO)_2_, 0.02 M CH_3_COONH_4_, and 5% DMSO. The MnO_2_ was deposited onto the graphene/Ni substrate using the electrochemical deposition method to fabricate MnO_2_/graphene/Ni electrodes. The input electric current per unit area of working electrode for the electrochemical deposition was the “current density”, expressed in mA·cm^−2^. The current densities of the electrochemical deposition were 1, 5, 10, and 15 mA·cm^−2^. The reaction time was 180 s. The samples were dried in a vacuum oven for 8 h at 60 °C. Finally, these composite electrodes were heated in a tube furnace at 250 °C with nitrogen gas for 2 h.

The deposited materials on the Ni foam were measured using an ultra-precise balance (RADWAG, XA 60/200/X). The balance could be scaled to a mass of five decimal digits in grams. The balance was settled on an optical shockproof table. The operation required great care and patience. The mass of Ni (1 cm × 1 cm) was 52.96 (±1.22) mg and the as-coated graphene was approximately 0.12 (±0.02) mg for a 10 min immersion. Graphene/Ni was utilized as the substrate and electrodeposition technology was applied to deposit the MnO_2_; the quality of the deposited MnO_2_ was related to the current density of the working electrode. The higher the applied current density, the higher the mass of the deposited MnO_2_. The current densities were 1 mA cm^−2^, 5 mA cm^−2^, 10 mA cm^−2^, and 15 mA cm^−2^, and the electrodeposition was for 180 s. Considering the error of mass weighing, the mass of the electrodeposited MnO_2_ was presented as 0.09 (±0.01) mg, 0.12 (±0.02) mg, 0.18 (±0.02) mg, and 0.25 (±0.03) mg. It is reproducible for the mass weighing of the very light deposited material. The mass load for each sample was calculated and is listed in Supplementary Materials, Table S1.

### 2.4. Characterizations 

A field-emission scanning electron microscope (FESEM, JEOL6330) with an acceleration voltage of 80 kV was utilized to examine the microstructures of the as-prepared MnO_2_/graphene/Ni electrodes. The structures of the prepared samples were examined using X-ray diffraction (XRD, Bruker, D8 ADVANCE) with Cu K_α_ radiation (λ = 1.5406 Å). X-ray photoelectron spectroscopy (XPS, Kratos Axis Ultra DLD) patterns were obtained using a monochromatic Al-anode X-ray gun. A binding energy of 284.6 eV for C was used to calibrate the charge-shifted energy scale. The spectra were deconvoluted for chemical identification using 100% Gaussian peaks. The Raman spectrum was determined using a Jobin-Yvon Lab Ram HR800 Raman spectroscope equipped with a 514.5 nm laser source.

### 2.5. Electrochemical Characterization of Single Electrode and Asymmetric Supercapacitor

All electrochemical measurements were carried out in 0.5 M Na_2_SO_4_ electrolyte. The electrochemical experiment was measured in a conventional three-electrode system using the MnO_2_/graphene/Ni foam composite (1.0 cm × 1.0 cm) as the working electrode, a Pt wire (1.0 cm × 1.0 cm) as the counter electrode, and a saturated Ag/AgCl electrode as the reference electrode.

Cyclic voltammetry (CV) and galvanostatic charging–discharging (GCD) were measured using a CHI 760D electrochemical workstation.

The specific capacitance (*C_s_*) could be obtained using the CV measurement with Formula (1) [23]:

(1) $$C_{s}=\int_{Va}^{Vb}\frac{\left|i\right|dV}{2m\nu \Delta V}$$

where *C_s_* is the specific capacitance (F g^−1^), *i* is the current (A), ν is the scan rate (V s^−1^), m is the mass of the electrode active material (MnO_2_) (g), and Δ*V* is the voltage range (V). In Formula (1), the *C_s_* obtained is divided by 2 because the area of the CV curve is calculated by a cycle of oxidation–reduction, which is also the cycle of charge–discharge. Moreover, the CV curve area of the graphene is significantly small, indicating that the specific capacitance of graphene is much less than that of MnO_2_. Therefore, we only considered the mass of MnO_2_ as the electrode active material mass (Supplementary Materials, Figure S1). 

Moreover, the specific capacitance (*C_m_*) was obtained according to the discharge curve of the GCD test using Formula (2):

(2) $$C_{m}=\frac{i\times \Delta t}{\Delta V\times m}$$

where *i* (A) is the discharge current, Δ*t* (s) is the discharge time, Δ*V* (V) is the discharging potential difference, and m (g) is the mass of the active material (MnO_2_). 

Electrochemical impedance spectra (EIS) were determined at an open-circuit voltage, with a bias of 10 mV for frequencies ranging from 100 kHz to 0.01 Hz, to test the electron transport properties.

Finally, an asymmetric supercapacitor with MnO_2_-graphene/Ni, obtained from 5 mA cm^−2^ of the electrodeposition current density for 180 s, as the positive electrode and graphene/Ni as the negative electrode was constructed, and electrochemical characterizations were carried out in electrolytes containing Na_2_SO_4_. The as-fabricated asymmetric supercapacitor cell was denoted as MnO_2_/graphene/graphene. Moreover, the equilibrium mass ratio of the positive electrode (MnO_2_) to the negative electrode (graphene) could be expressed according to Formula (3):

(3) $$\frac{m_{+}}{m_{-}}=\frac{\Delta E_{+}\times C_{+}}{\Delta E_{-}\times C_{-}}\;$$

where m_+_ is the mass (g) of the active material (MnO_2_) in the positive electrode, m_−_ is the mass (g) of graphene in the negative electrode C is the specific capacitance (F g^−1^), and ΔE is the potential window of the charge and discharge process (V). Based on Formula (3), the mass ratio of the two electrodes, m_+_/m_−_, is approximately 1.6/1.

Furthermore, the energy density, E (Wh kg^−1^), and the power density, P (W kg^−1^), could be estimated from the discharge curve using Formulas (4) and (5):

(4) $$E=\frac{1}{2}C_{m}\Delta V^{2}$$

(5) $$P=\frac{E}{\Delta t}$$

## 3. Results and Discussion 

### 3.1. Characterization of the MnO_2_-Based Electrode Materials

**Scheme 1:**
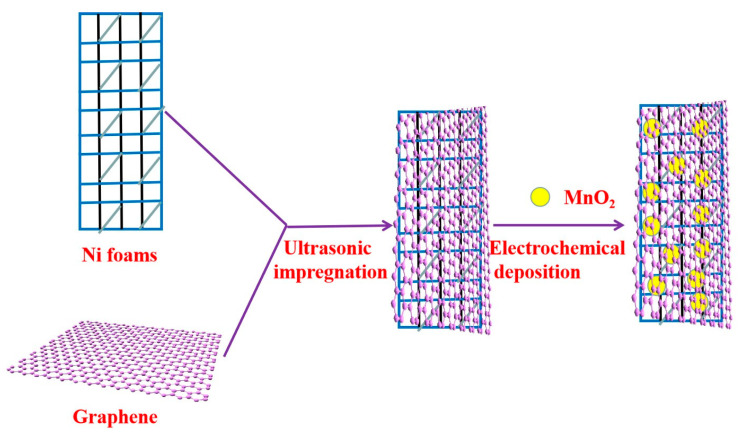
Schematic illustration of the fabrication procedures for the MnO_2_/graphene/Ni composite using a facile method.

Due to ultrasonic cavitation, ultrasonic-assisted impregnation generated special physical states, such as a higher temperature and higher pressure locally than without the apparatus, which can increase graphene loading and improve the dispersity, thus enhancing the activity. The deposition technology followed the steps to grow nucleation sites and then ions reacted to produce the deposition material on the substrate. In this case, MnO_2_ obtained by oxidation of Mn^2+^ from manganese acetate was adsorbed onto graphene coated on the Ni foam. The MnO_2_/graphene/Ni composite was fabricated using a facile method, as shown in Scheme 1.

**Figure 1:**
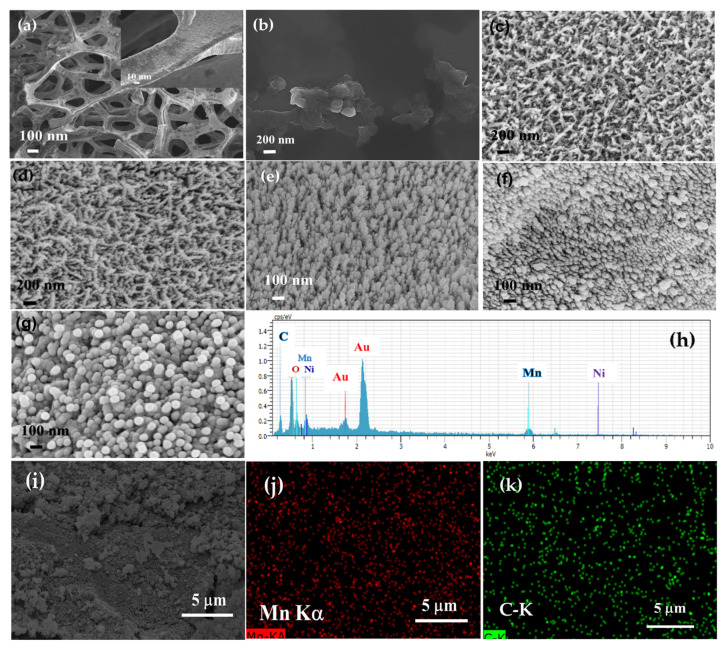
FESEM images of Ni foams (**a**), graphene/Ni foams (**b**), MnO_2_/graphene/Ni electrode (1 mA cm^−2^ (**c**)), MnO_2_/graphene/Ni electrode (5 mA cm^−2^ (**d**)), MnO_2_/graphene/Ni electrode (10 mA·cm^−2^ (**e**)), MnO_2_/graphene/Ni electrode (15 mA cm^−2^ (**f**)), and MnO_2_/graphene/Ni electrode with the current of 1 mA cm^−2^ for 30 min deposition (**g**). EDX spectrum of MnO_2_/graphene/Ni electrode with current of 1 mA cm^−2^ for 30 min deposition (**h**). FESEM image of MnO_2_/graphene/Ni electrode and both Mn and C mapping of MnO_2_/graphene/Ni material (**i**–**k**).

SEM was used to study the morphologies of the prepared samples. Figure 1a shows an SEM image of the nickel foams. The insert image shows that such a smooth substrate not only supplies the platform for the deposition of graphene and MnO_2_, but also facilitates the fast channel of electrolytes to the electrode. It could be observed that the pore distribution of nickel foams was approximately between 100 μm and 300 μm, in which the porous structure of the nickel foams had a higher surface area. Therefore, the fabricated electrode and electrolyte had a greater contact area to allow for a higher super capacitance. The SEM image of the graphene/Ni foam composite with 20,000× magnification is shown in Figure 1b. It could be observed that graphene prepared by electrolytic-stripping had a sheet nanostructure. However, the coated graphene/Ni was obtained in an oven at 100 °C for 1 h to adhere the graphene onto the nickel foam, thus the surface exhibited densely packed but porous morphology. It was indicated that the number of layers of graphene can be revealed by the Raman shift of the 2D band at approximate 2700 cm^−1^ of the Raman spectra. The 2D band for an extremely few layer of grsaphene exhibits sharper and a more symmetrical shape [24]. For the graphene/Ni in the present study, the 2D band at 2700 cm^−1^ was not sharp enough, indicating that the number of graphene layers obtained was relatively high (Supplementary Materials, Figure S2).

Figure 1c–f shows SEM images of MnO_2_/graphene/Ni composites with different constant currents for 10 min. The diameter of the MnO_2_ nanoparticles ranged from 10 nm to 15 nm, with a constant current of 1 mA cm^−2^. The diameter of MnO_2_ nanoparticles ranged from 10 nm to 20 nm, with a constant current of 5 mA cm^−2^. The diameter of MnO_2_ nanoparticles was about 30 nm, with a constant current of 10 mA cm^−2^. The diameter of MnO_2_ nanoparticles was about 50 nm, with a constant current of 15 mA cm^−2^. These results indicated that the MnO_2_ was homogeneously dispersed and uniformly deposited on the skeleton of the graphene/Ni composite. Actually, the thickness of MnO_2_ varied in different places of the sample. It could be that only scale 200 μm was flat. The diameters of MnO_2_ nanoparticles increased with the increase in the constant current. Figure 1g presents an SEM image of MnO_2_/graphene/Ni composite with a current of 1 mA·cm^−2^ for a 30 min deposition. When the deposition time was longer, the particle size increased continuously, which greatly reduced the surface area of the particles, leading to a decrease in the contact area of the electrolyte. C, Mn, O, Au, and Ni were observed in the EDX spectrum (based on the SEM image of Figure 1g) of the MnO_2_/graphene/Ni composite (prepared at 1 mA cm^−2^, 30 min deposition), which were attributed to graphene, MnO_2_, Au, and Ni. However, Au derived from the Au-coating of the sample for SEM image measurement, as shown in Figure 1h. Furthermore, the Mn and C in the electrode dispersed well in the material (Figure 1i,j,k), indicating that MnO_2_ has good dispersibility in the MnO_2_/graphene/Ni electrode. TEM was also utilized to identify the images of MnO_2_ and MnO_2_/graphene. It could be clearly observed that the prepared MnO_2_ structure was loosely arranged. The wrinkle-like morphology for graphene could be observed. Considering the MnO_2_/graphene was prepared by the deposition process, the graphene could restack, resulting in excessive surface agglomeration (Supplementary Materials, Figure S3).

**Figure 2:**
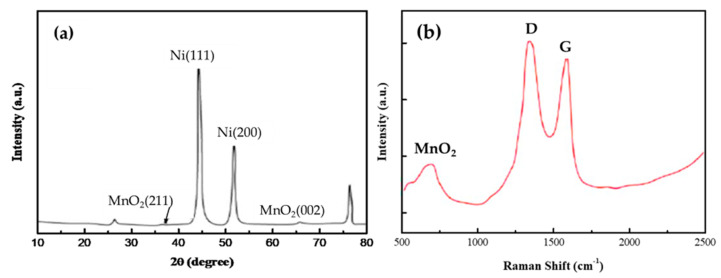
The XRD patterns of the MnO_2_/graphene/Ni electrode (**a**) and Raman spectra of the MnO_2_/graphene/Ni electrode (**b**).

The crystal structures of the prepared samples were characterized by XRD, as shown in Figure 2a. The obvious characteristic peaks at 44.5°, 51.7°, and 76.5° were attributed to the (111), (200), and (220) crystal planes of the Ni foams. The peak at 26.4° belonged to the diffraction peak of the carbon material, which corresponded to the (002) crystal plane of the graphene. Two weak peaks at 37.5° and 65.6° were attributed to the (211) and (002) diffraction peaks of α-MnO_2_ (JCPDS Card NO.44-0141) [25]. The results could be further confirmed by the Raman spectra of the MnO_2_/graphene/Ni composite, as shown in Figure 2b. The D band is a common feature for sp^3^ defects or disorder in carbon, and the G band provides useful information on in-plane vibration of sp^2^-bonded carbon atoms in a 2-D hexagonal lattice. The D band (~1345 cm^−1^) and G band (~1578 cm^−1^) were assigned to the characteristic bands of graphene. The G band represents the in-plane vibration of the sp^2^ carbon atoms. The D band is related to the breathing mode of κ-point phonons of the A1g symmetry [26]. The relative intensity ratio of the D-band to G-band, defined as R = I_D_/I_G_, represents the structural regularity of graphene. The R-value was proposed to be an indication of disorder in graphene. The smaller the R value, the greater the regularity is. As seen from Figure 2b, the R-value is approximately 1.15, demonstrating an irregular structure in graphene compare with our previous study [27]. A broad (observed at ~630 cm^−1^) peak could be attributed to the symmetric stretching vibrations of Mn–O.

**Figure 3:**
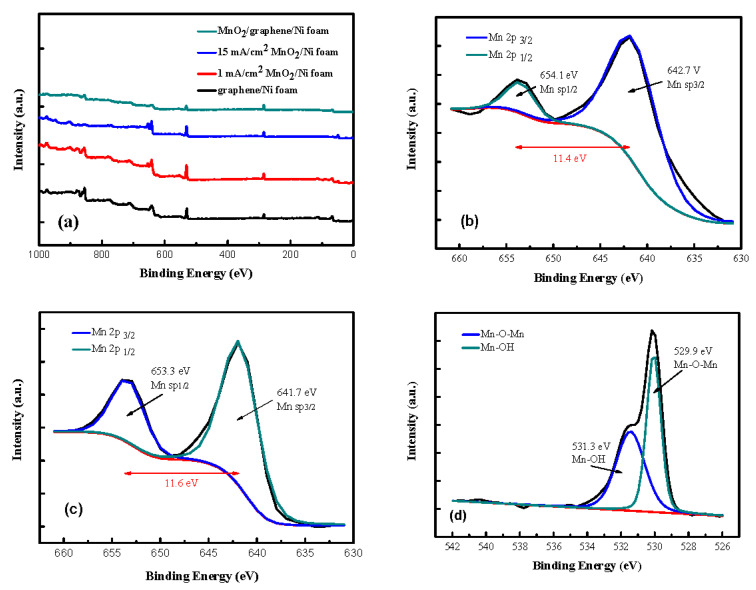

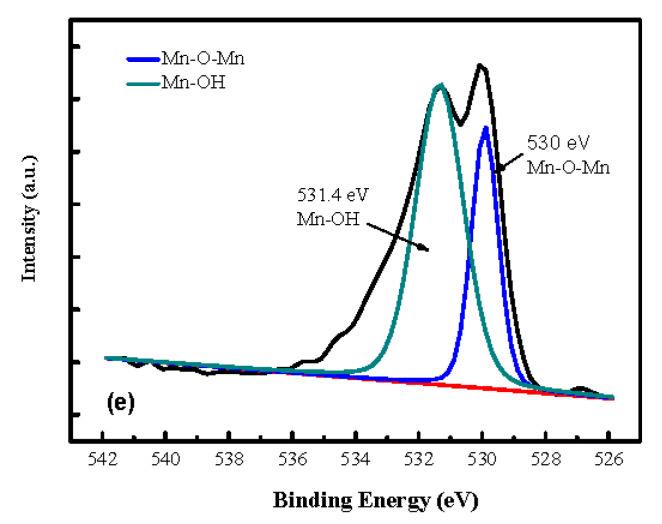
(**a**) The XPS full-spectrum of the MnO_2_/graphene/Ni electrode; (**b**,**c**) high-resolution Mn(2p) spectra of the MnO_2_/graphene/Ni electrode obtained at 1 mA cm^−2^ and at 15 mA cm^−2^ electrodeposition; and (**d**,**e**) high-resolution O(1s) spectra of the MnO_2_/graphene/Ni electrode obtained at 1 mA cm^−2^ and at 15 mA cm^−2^ electrodeposition.

XPS was used to determine the changes in the surface charge states. The full spectra of MnO_2_/graphene/Ni composites are shown in Figure 3a. Clearly, graphene/Ni foam contained two elements (C and O). After electrochemical deposition, three element signals (C, O, and Mn) could be observed, which indicates that MnO_2_ was successfully deposited on the graphene/Ni foam. The peak at 654.1 eV was attributed to Mn 2p. The peaks at 285.7 eV (C-O), 284.8 eV (C-C sp^3^), and 284.2 eV (C=C sp^2^) were similar to those in the literature [28,29,30]. The coexistence bonds of C-C (sp^3^) and C=C (sp^2^) were due to the appearance of larger graphite fragments during the process of graphite stripping. The high-resolution Mn(2p) of the MnO_2_/graphene/Ni foam composite was amplified for fitting analysis at different current densities. The peaks at 654.1 eV and 653.3 eV were attributed to Mn 2p_1/2_, while the peaks at 642.7 eV and 641.7 eV were related to Mn 2p_3/2_, as seen in Figure 3b,c. The Mn 2p peak was fitted into two peaks (2p_1/2_ and 2p_2/3_) at 654.1 eV and 642.7 eV, respectively, indicating that Mn was represented in the chemical state of Mn^4+^. The peak values were 653.3 eV and 641.7 eV (Figure 3c), implying that they were not only tetravalent but also bivalent and trivalent manganous oxide. The energy separation was about 11.4 eV, which was related to α- MnO_2_ [31], suggesting that MnO_2_ was successfully formed. To obtain pure MnO_2_, the optimum current density was fixed at 1 mA·cm^−2^ in the following experiment. Figure 3d,e show high-resolution O(1s) spectra of MnO_2_/graphene/Ni composites at different current densities. The binding energy value was 529.9 eV and 531.3 eV (Figure 3d), which was attributed to Mn-O-Mn and Mn-OH, respectively. The Mn-O-Mn and Mn-OH presented at 530 eV and 531.4 eV, and are shown in Figure 3e, indicating that the structure of manganese was complex. It was found that manganese dioxide had a different composition according to the constant current density. When the current density increased, the signal peak of Mn-OH increased to decrease the content of manganese oxide.

### 3.2. Electrochemical Properties of Single Electrodes

**Figure 4:**
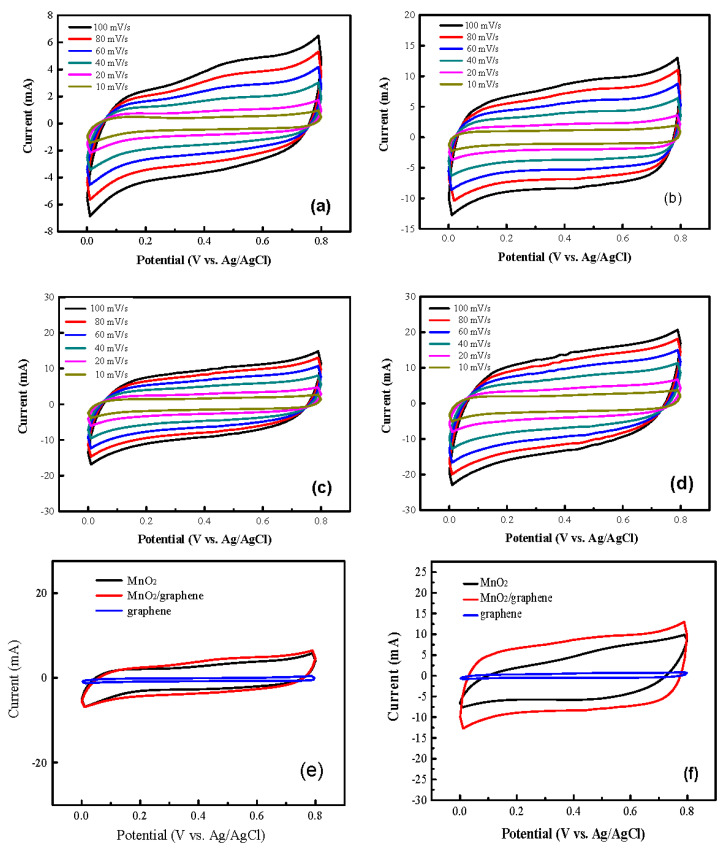

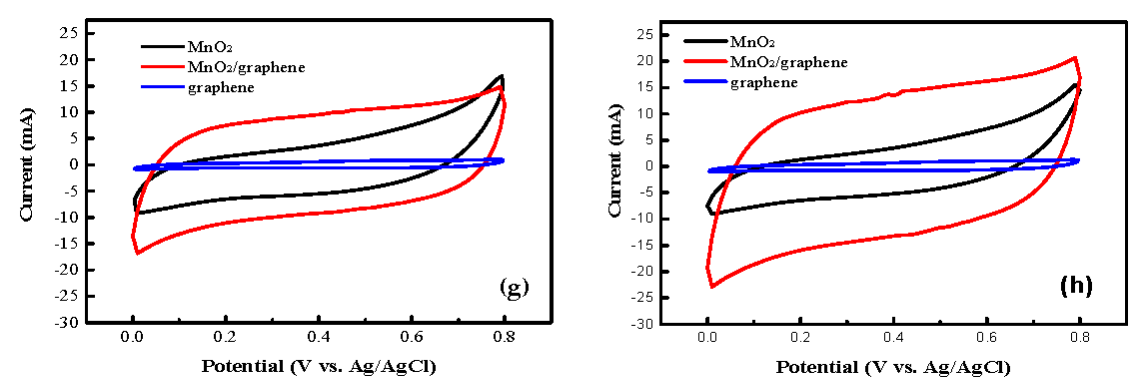
The CV curves of MnO_2_/graphene/Ni electrodes prepared from different current densities using the electrodeposition method: (**a**) 1 mA cm^−2^, (**b**) 5 mA cm^−2^, (**c**) 10 mA cm^−2^, and (**d**) 15 mA cm^−2^. The CV curves of graphene/Ni, MnO_2_/Ni, and MnO_2_/graphene/Ni electrodes: (**e**) 1 mA cm^−2^, (**f**) 5 mA cm^−2^, (**g**)10 mA cm^−2^, and (**h**) 15 mA cm^−2^.

In the study, Ni foam with high conductivity was used as the current collector. The CV characteristic curve was narrow and small, indicating a very small specific capacitance (Figure S4). Figure 4a–d show the CV curves of MnO_2_/graphene/Ni electrodes at different current densities. As the scanning rate increased from 10 mV s^−1^ to 100 mV s^−1^, the shapes of the CV curves changed slighty for all the samples, implying they possessed excellent electrochemical reversibility and stability. The specific capacitances of MnO_2_/graphene/Ni foam prepared at different scanning rates under different current densities are presented in Table 1. Each experimental data point was measured several times and calculated using a statistical analysis method; the mean and standard error of the mean were obtained; and the mean was taken from three significant figures.

It was clearly observed that the specific capacitance of the MnO_2_/graphene/Ni electrode decreased as the current density increased. The highest specific capacitance came from the lowest current density. At lower scan rates, the diffusion of Na^+^ from the electrolyte could transport ions to the interior and interface between MnO_2_ and graphene, leading to a completed reaction and process. At a high scan rate, Na^+^ is pushed onto the outer surface layer of the electrode, where effective interaction between ions and electrodes is reduced. Therefore, the specific capacitance would be decreased. The specific capacitance of the electrodes can be calculated according to Formula (1). 

From Formula (1), ν was lower and *C_s_* was higher. By increasing ν, the current became larger and the ions in the electrolyte did not have enough time to diffuse into the electrode materials. The capacitance performance of MnO_2_/Ni and MnO_2_/graphene/Ni electrodes were investigated using circle voltammetry at a scan rate of 100 mV s^−1^ for different current densities using the electrodeposition method, as seen in Figure 4e–h. Since the specific capacitance was directly proportional to the area of the CV curves, the results showed that the CV area of the MnO_2_/graphene/Ni foam electrodes was much larger than that of the MnO_2_/Ni foam electrode. Moreover, the electrodeposition current density was high, causing the deposited mass of MnO_2_ to be large. However, the CV characteristic showed that MnO_2_/graphene/Ni did not change much, demonstrating that they were almost quasi-rectangular shapes. It was obvious that graphene could improve the conductivity of MnO_2_. In addition, due to the increase of MnO_2_ mass deposition on MnO_2_/Ni by increasing with the current density of the electrodeposition, the CV characteristic deviated from the rectangular shape (Figure 4g,h). As seen in Figure 4e–h, due to the fact that the area of the CV curve of graphene was much less than that of MnO_2_, in this work, only the mass of MnO_2_ was used as the active material mass.

The choice of aqueous electrolyte was based on the size of the hydrated cations and anions, and on the mobility of ions [32]. In addition, the corrosiveness of the electrolyte with regard to the electrode must be considered. The Na_2_SO_4_ solution was approximately a neutral electrolyte, which is friendly to the environment and was utilized as the electrolyte in this study. The MnO_2_/graphene/Ni electrodes showed a quasi-rectangular shape in the CV curves, indicating the capacitance characteristics of the MnO_2_ deposited onto the Ni foam electrode [33]. The CV curve for the MnO_2_/graphene/Ni electrode in the Na_2_SO_4_ electrolyte was unlike that expected from an EDLC; the CV characteristic curve for an EDLC shows a nearly ideal rectangle [34].

In this article, the capacitances are compared with other calculation methods, which depends on the mass ratio of graphene and the MnO_2_ of the composite electrode. It was calculated and the specific capacitance (*C_s_*) is listed based on $C_{s}=\int \frac{\left|i\right|dV}{2m\nu \Delta V}\;$; the total loaded mass (MnO_2_+graphene, M) as active materials was calculated by $C_{s}^{\prime }=\int \frac{\left|i\right|dV}{M\nu \Delta V}$ to obtain the specific capacitance (*C_s_*′) and areal capacitance *C*_*s*A_′ based on *C_s_*′, which were also calculated for the comparison (Supplementary Materials, Table S2). In this study, Ni foam was used as the substrate material and this material has a porous structure. The pores have a significant influence on the capacitance, thus it is not suitable to discuss the volume capacitance.

The GCD test for MnO_2_/graphene/Ni electrodes obtained from different electrodeposition current densities were examined at 1 A g^−1^. The longer discharge time of the MnO_2_/graphene/Ni electrode prepared at an electrodeposition current density of 5 mA cm^−2^ exhibited a capacitance higher than that of the other electrodes, which is consistent with the results obtained from the CV characterizations. It could be seen that the MnO_2_/graphene/Ni electrode exhibited highly linear and almost symmetrical triangular curves, revealing that the IR potential drop for MnO_2_/graphene/Ni is less noticeable. However, the GCD curve was slightly bent at the end of the discharge curve for the pseudo-capacitor material. This was mainly caused by the redox reaction inside the material. Thus, the graphene composite MnO_2_ electrode has good electrochemical performance (Supplementary Materials, Figure S5).

**Figure 5:**
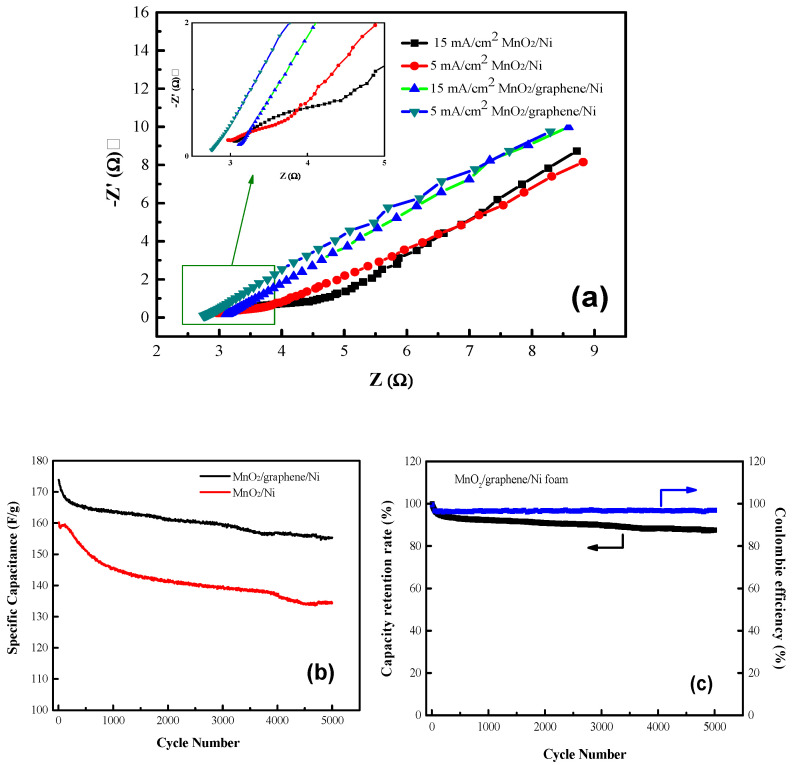
Nyquist plots of the electrodes (**a**), the cycling characteristics of the two electrodes under the current density of 5 A g^−1^ (**b**), and the coulombic efficiency of the MnO_2_/graphene/Ni electrode (**c**).

To study the electrochemical mechanism for the MnO_2_ electrode materials obtained from various electrodeposition conditions, they were subjected to EIS analysis, as shown in Figure 5a. EIS measurements were taken in a frequency range from 100 kHz to 0.01 Hz. 

The results are displayed using Nyquist plots, which were divided into three different regions, as follows.

In the high frequency region, the intercept at the real axis (Z_0_) represents the equivalent series resistance (ESR), including the ionic resistance of the electrolyte, the inherent resistance of the substrate, and the contact resistance of the active material/current collector interface [35]. The span of the semicircular arc in the mid–high frequency region represents the charge transfer resistance (R_ct_) at the electrode/electrolyte interface [36]. The slope in the low frequency region was attributed to semi-infinite Warburg impedance, which played an important role in the frequency dependence of diffusion/transport for electrolyte ions in the holes of the electrode [37]. If the impedance graph increases sharply and tends to become a vertical line, the characteristic of a pure capacitance behavior is indicated. The impedance values of the electrodes are summarized in Table 2. In the table, each experimental data point was measured several times and calculated using a statistical method; the mean and standard error of the mean were obtained; and the mean was taken with two significant figures.

At high frequencies, the intercepts (R_E_) for the curve and real axis for the MnO_2_/graphene/Ni electrode (prepared at 5 mA cm^−2^ current density of the electrodeposition), the MnO_2_/graphene/Ni electrode (prepared at 15 mA cm^−2^ current density of the electrodeposition), the MnO_2_/Ni electrode (prepared at 5 mA cm^−2^ current density of the electrodeposition), and the MnO_2_/Ni electrode (prepared at 15 mA cm^−2^ current density of the electrodeposition) were determined to be 2.8 Ω, 3.1 Ω, 3.0 Ω, and 3.1 Ω, respectively. In particular, the MnO_2_/graphene/Ni electrode obtained from the electrodeposition at a current density of 5 mA cm^−2^ showed the smallest equivalent resistance, demonstrating that the electrode had better conductivity. Moreover, the as-obtained MnO_2_/graphene/Ni electrode (5 mA cm^−2^) showed the smallest semicircular arc-shaped impedance (0.15 Ω) in the high–medium frequency region, indicating that the charge transfer resistance (R_ct_) for the electrode was extremely low and that the ion diffusion path was very short. 

In the low-frequency region, the MnO_2_/graphene/Ni electrode showed a straight line with a steeper slope, indicating that the capacitance performance was very close to that of an ideal supercapacitor [38]. Additionally, in this region, the slope of the impedance curve for the electrode composited without graphene was not as steep (less steep) as that for the electrode with a graphene buffer layer. In addition, the R_N_ at 2.8 Ω and 3.1 Ω for MnO_2_/graphene/Ni was smaller than those of 3.4 Ω and 3.9 Ω for MnO_2_/Ni electrodes (Table 2). The sum of the impedance values of the three regions for each electrode prepared using different electrodeposition methods as described above is also listed in Table 2. The results also showed that R_t_ at 5.8 Ω and 6.6 Ω for MnO_2_/graphene/Ni electrodes was smaller than those of 7.0 Ω and 8.5 Ω for the MnO_2_/Ni electrodes. These results may be due to the relatively better dispersion of using graphene in the MnO_2_/graphene/Ni electrode.

In this study, the MnO_2_/graphene/Ni electrode obtained using a 5 mA cm^−2^ current density electrodeposition exhibited an extremely low impedance, which was attributed to the better homogeneity and nanostructure of the composites grown on the nickel foam. In addition, the MnO_2_/graphene/Ni (5 mA cm^−2^) electrode exhibited an equivalent series resistance (R_E_) that was lower than that of the other electrodes. This result further showed that the MnO_2_/graphene/Ni (5 mA cm^−2^) electrode had faster kinetics compared to the other three electrodes, which is beneficial in improving the capacitance performance of the composite material, especially at high charge/discharge rates for the supercapacitor [39].

The MnO_2_/graphene sample had a clear hysteresis loop, showing highly interconnected holes, and the material had an open-wide structure. The BET specific surface areas for the MnO_2_ and MnO_2_/graphene materials were 158.5 m^2^·g^−1^ and 179.2 m^2^·g^−1^, respectively, and the pore sizes were 3.7 nm and 7.8 nm, respectively. MnO_2_/graphene showed the largest BET specific surface area, which is attributed to the loosely arranged structure (Supplementary Materials, Figure S6 and Table S3). The electrode possesses a better pore structure and allows the Na_2_SO_4_ electrolyte to be easily adsorbed on the electrode, which facilitates migration and diffusion, resulting in a larger CV curve area and a higher specific capacitance value.

Figure 5b shows the cycling characteristics of the two electrodes under a current density of 5 A g^−1^. It is clear that the specific capacitance of the MnO_2_/Ni electrode was maintained at 84%, while the capacitance retention rate of the MnO_2_/graphene/Ni electrode increased to 90% after 5000 cycles. It is indicated that the introduction of a graphene layer can improve the poor conductivity of MnO_2_ and increase the reversibility of both absorbing and desorbing electrons. The graphene layer not only improves the whole capacitance but also increases the retention rate of the capacitance. Another reason for this may be that a good adhesion between graphene and MnO_2_, as well as between graphene and the Ni substrate, is presented. Here, graphene is considered as a buffer layer. Figure 5c shows the coulombic efficiency of the MnO_2_/graphene/Ni electrode. The coulombic efficiency is the ratio of the charge passing through an electrode to the reaction charge on the electrode. From the figure, it can be observed that the coulombic efficiency remained at 96% after 5000 cycles during the charge and discharge process. The charge transfer efficiency of the electrode and the decrease in the stored charge of the battery due to the aging effect during the actual charge and discharge process can thus be known. 

**Figure 6:**
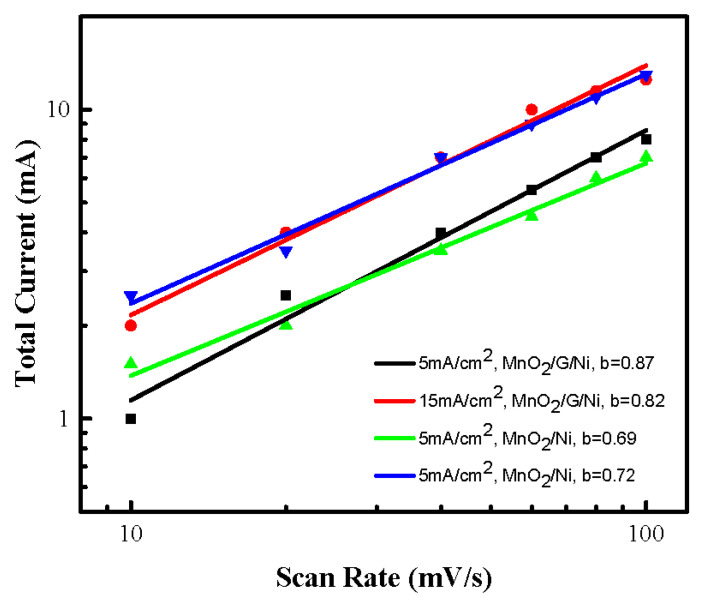
The **b** parameter of the MnO_2_/Ni and MnO_2_/graphene/Ni electrodes obtained from different electrodeposition conditions.

The total current (*i*(*v*)) of the CV measurement at scan rates for the composite material consisted of two parts. One part concerned the current associated with the EDLC at the electrolyte interface or the initial fast Faraday reaction on the exposed electrode surface (*i_cap_*). The other part concerned the current associated with the slow diffusion control process (*i_dif_*). The capacitance contribution and diffusion control contribution can be calculated according to Formula (6) [40].

(6) $$i\left(v\right)=i_{cap}+i_{dif}=a\times \nu^{b}$$

where *ν* is the scan rate, *i*(*v*) is the total current of the CV measurement, and a and b are variable parameters. The b value can be estimated from the slope of the log(*i*(*v*)) and log(*ν*). Figure 6 shows the b parameter of the MnO_2_ /Ni and MnO_2_/graphene/Ni electrodes obtained from different electrodeposition conditions. 

It can be observed that the MnO_2_/graphene/Ni electrodes indicated that 0.8 < b < 1 electrode was considered to be a pseudocapacitive material with a main capacitance storage, while the MnO_2_/Ni electrodes showed “0.5 < b < 0.8” as exhibiting the main Faraday (battery type) behavior [40,41]. b = 1 indicates EDLC, as well as the slope in the Nyquist plot 90°, while b = 0.5 indicates battery-type, as well as the slope in the Nyquist plot 45°. The “b parameter” of the four different electrodes were all greater than 0.5, which obviously reveal that the final element will not be a hybrid cell.

### 3.3. MnO_2_/Graphene//Graphene Asymmetric Supercapacitor

Asymmetric supercapacitors were fabricated as devices for studying capacitive performance for practical applications. MnO_2_/graphene/Ni was used as the positive electrode, graphene deposited on nickel foams was used as the negative electrode, and 0.5 M Na_2_SO_4_ aqueous solution was used as the electrolyte. 

During the charge–discharge of a supercapacitor, a water-based electrolyte must avoid the generation of hydrogen and oxygen or other redox side reactions due to the electrolysis of water affecting the performance of the device. These phenomena can be revealed by their CV characteristics and a suitable operating potential window would be obtained.

**Figure 7:**
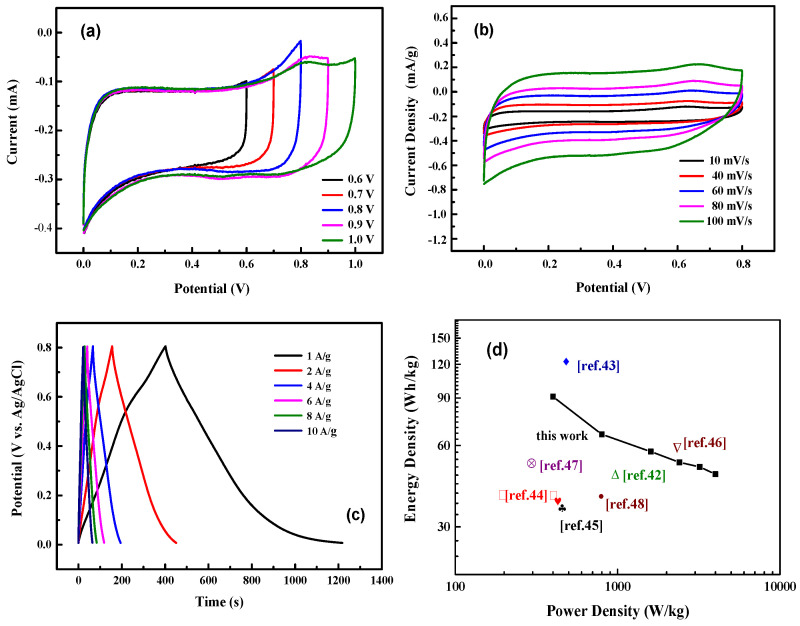
The electrochemical performance of the as-assembled MnO_2_/graphene//graphene asymmetric supercapacitor: (**a**) the CV curves of the as-obtained MnO_2_/graphene//graphene asymmetric supercapacitor scan at various scan rates (**b**); the GCD test of the assembled asymmetric supercapacitor (**c**); and the Ragone plots of the MnO_2_/graphene//graphene asymmetric supercapacitor (**d**).

Figure 7 shows the electrochemical performance of the as-obtained MnO_2_/graphene//graphene asymmetric supercapacitor. The CV curves of the as-prepared asymmetric supercapacitor at a scan rate of 50 mV s^−1^ for different potential windows are shown in Figure 7a. When the potential window was from 0.6 V to 0.8 V, the CV curve maintained an approximately symmetrical shape, indicating that this asymmetric supercapacitor still has a good reversible cycle at 0.8 V. However, when the potential window was 0.9 V or 1.0 V, the CV curve exhibited a weak redox peak and the curves also showed an asymmetric shape, which means that, under this potential window, the electrochemical characteristics of the device are irreversible and the energy storage performance cannot be fully exerted. There was no significant influence of the CV curve-shape that occurred at a high scan rate of 100 mV s^−1^ (Figure 7b), indicating that the device at this scan rate maintains a high stability. 

The GCD test of the assembled MnO_2_/graphene//graphene asymmetric supercapacitor was characterized at various current densities and is shown in Figure 7c so as to calculate the specific capacitance (*C_m_*) of the cell using Formula (2). At current densities of 1 A g^−1^, 2 A g^−1^, 4 A g^−1^, 6 A g^−1^, 8 A g^−1^, and 10 A g^−1^, the specific capacitance (*C_m_*) of the MnO_2_/graphene/graphene device exhibited 1025 F g^−1^, 740 F g^−1^, 640 F g^−1^, 587 F g^−1^, 556 F g^−1^, and 531 F g^−1^, respectively.

The energy density and power density of the asymmetric supercapacitor were calculated based on Formulas (4) and (5) according to the data obtained from the GCD characterization, as the Ragone plots in Figure 7d show. A comparison of the MnO_2_/graphene//graphene asymmetric supercapacitor with those recently published in the literature is summarized in the figure. In the MnO_2_/graphene//graphene asymmetric supercapacitor, at a power density of 400 W kg^−1^, the energy density was as high as 91 Wh kg^−1^ at 1 A g^−1^, and at a power density of 800 W kg^−1^, it remained at 66 Wh kg^−1^ at 2 A g^−1^, which is higher than that of the studied asymmetric supercapacitors in the literature [42,43,44,45,46,47,48]. However, the devices referred to in Table S4 have been checked: they have the same energy storage mechanism and are not hybrid devices (Supplementary Materials, Table S4).

## 4. Conclusions

In summary, we employed a promising strategy for fabricating a high-performance MnO_2_/graphene/Ni electrode. The electrode prepared at a current density of 1 mA cm^−2^ using the electrodeposition method with a structure of a 3D framework and a 2D graphene layer exhibited a higher power output, where the highest capacitance (292.8 F·g^−1^) was obtained in a 0.5 M Na_2_SO_4_ solution. The Ni foams with a 3D framework provided a larger surface area, increasing the deposition sites for introducing MnO_2_. Graphene was considered as a buffer layer between the MnO_2_ and Ni foams to improve the electrical conductivity and interface contact. The coulombic efficiency remained at about 96% after 5000 cycles during the charge and discharge process. Therefore, the technology not only increased specific capacitance but also improved mechanical stability. A MnO_2_/graphene/graphene asymmetric supercapacitor was assembled and exhibited both a high-energy density of 91 Wh kg^−1^ and an excellent power density of 400 W kg^−1^ at 1 A g^−1^. We expect that this simple method may provide a convenient route for the large-scale production of MnO_2_/graphene/Ni electrodes with a high capacitance for high-energy storage in future.

## Figures and Tables

**Table 1:** The specific capacitance of MnO_2_/graphene/Ni foam at different scanning rates under different current densities.

Scan Rate	Specific Capacitance (F g^−1^)			
	1 mA/cm^2^	5 mA/cm^2^	10 mA/cm^2^	15 mA/cm^2^
100 mV/s	202.9 (±1.9)	155.8 (±1.4)	76.7 (±0.6)	52.4 (±0.8)
80 mV/s	205.7 (±1.7)	141.6 (±1.5)	83.5 (±0.7)	57.5 (±0.6)
60 mV/s	214.3 (±1.4)	170.0 (±1.1)	91.4 (±0.7)	63.5 (±0.9)
40 mV/s	230.6 (±1.8)	181.8 (±1.8)	114.2 (±0.8)	71.6 (±0.8)
20 mV/s	261.6 (±2.3)	202.0 (±1.9)	122.9 (±1.4)	86.3 (±0.6)
10 mV/s	292.8 (±2.1)	222.3 (±2.3)	142.0 (±1.1)	99.6 (±0.9)

**Table 2:** The impedance value of MnO_2_/Ni and MnO_2_/graphene/Ni electrodes.

Samples	*R_t_* (Ω)	*R_E_* (Ω)	*R_CT_* (Ω)	*R_N_* (Ω)
MnO_2_/graphene/Ni (5 mA/cm^2^)	5.8(±0.4)	2.8(±0.2)	0.15(±0.1)	2.8(±0.1)
MnO_2_/graphene/Ni (15 mA/cm^2^)	6.6(±0.5)	3.1(±0.2)	0.35(±0.1)	3.1(±0.2)
MnO_2_/Ni (5 mA/cm^2^)	7.0(±0.4)	3.0(±0.1)	0.64(±0.1)	3.4(±0.2)
MnO_2_/Ni (15 mA/cm^2^)	8.5(±0.7)	3.1(±0.2)	1.5(±0.2)	3.9(±0.3)

## Supplementary Materials

The following are available online at https://www.mdpi.com/article/10.3390/nano11102736/s1, Table S1: The mass of graphene and MnO_2_ loaded on the MnO_2_/graphene/Ni sample by electrodeposition at different current densities, Table S2: The specific capacitances (*C_s_*, *C_s_*’ and *C*_*s*A_) of MnO_2_/graphene/Ni foam prepared at different electrodeposition current densities obtained from CV curves under various scan rates, Table S3: The powder properties of MnO_2_ and MnO_2_/graphene materials, Table S4: A comparison of the energy density and power density for the MnO_2_//carbon system asymmetric supercapacitor in this work with the literature, Figure S1: (**a**) CV curves of 0.12 mg loaded graphene/Ni at different scan rates; (**b**) CV curves of MnO_2_, MnO_2_/graphene/Ni and graphene/Ni (Ni: 54.35 mg, MnO_2_: 0.09 mg, and graphene: 0.12 mg), Figure S2: The Raman spectra of as–produced graphene, Figure S3: The TEM analysis for the as-obtained MaO_2_ (**a**) and MnO_2_/graphene material (**b**), Figure S4: The CV characteristic curves of blank Ni foam at different scan rates, Figure S5: The GCD test for MnO_2_/graphene/Ni electrodes obtained from different electrodeposition current densities examined at 1 A g^−1^, Figure S6: BET specific surface area and pore size distribution analysis for MnO_2_ and MnO_2_/graphene materials. (**a**) N_2_ isotherm adsorption-desorption analysis and (**b**) pore distribution analysis.
