# Supplementary material for: Facile Fabrication of MnO2/Graphene/Ni Foam Composites for High-Performance Supercapacitors
Source: Nanomaterials (Basel). 2021 Oct 15;11(10):2736. doi: 10.3390/nano11102736 (PMC8537046; doi:10.3390/nano11102736)
Supplement: Supplementary file 1 [file nanomaterials-11-02736-s001.zip › nanomaterials-1413845-supplementary.pdf]

nanomaterials

MDPI

Supplementary Materials

Facile Fabrication of MnO_2/Graphene/Ni Foam Composites for High-Performance Supercapacitors

Rui Liu [1], Rui Jiang [1], Yu-Han Chu [2] and Wein-Duo Yang [2,*]

[1] Center of Pharmaceutical Engineering and Technology, School of Pharmacy, Harbin University of Commerce, Harbin 150076, China; liur@hrbcu.edu.cn (R.L.); liurui19992007@163.com (R.J.)
[2] Department of Chemical and Materials Engineering, National Kaohsiung University of Science and Technology, Kaohsiung 80778, Taiwan; churester@gmail.com
* Correspondence: ywd@nkust.edu.tw; Tel.: +886-7-3814526 (ext. 15116)

Table S1. The mass of graphene and MnO_2 loaded on the MnO_2/graphene/Ni sample by electrodeposition at different current densities.

	Blank Ni foam (mg)	Graphene loaded (mg)	MnO_2 loaded (mg)	Total loaded Weight (mg)	Ni: graphene: MnO_2 (mass ratio)
1 mA/cm^2	51.25(±2.56)	**0.12(±0.01)**	**0.09(±0.01)**	**0.21(±0.02)**	1: 0.002: 0.002
5 mA/cm^2	55.62(±2.22)	0.15(±0.02)	0.12(±0.02)	0.27(±0.04)	1: 0.003: 0.002
10 mA/cm^2	50.62(±3.50)	0.13(±0.01)	0.18(±0.02)	0.31(±0.03)	1: 0.003: 0.004
15 mA/cm^2	54.35(±2.72)	0.14(±0.02)	0.25(±0.03)	0.39(±0.05)	1: 0.003: 0.005

0.12(±0.01) mg, 0.09(±0.01) mg and 0.21(±0.02) mg were obtained by statistical analysis method as follows.

For example, the MnO_2/graphene/Ni foam electrode prepared at 1 mA/cm^2, the mass of graphene loaded was measured 5 times by ultra-precise balance. The mass of loaded graphene was 0.10 mg, 0.12 mg, 0.13 mg, 0.13 mg, and 0.14 mg, respectively. The data are calculated by statistical method as follows.

The experimental data is expressed as follows:

$$x = \bar{X} \pm \sigma_{\bar{x}}$$

x: Results of multiple mass weighing

$\bar{X}$: mean of measured data

$\sigma_{\bar{x}}$: standard error of the mean

$\bar{X}$= (0.10 + 0.12 + 0.13 + 0.13 + 0.14) ÷ 5

= 0.12

For a limited number of mass weighing, n= 5

$$\sigma_{\bar{x}} = \sqrt{\frac{\sum d_i^2}{n(n-1)}}$$

$$d = x - \bar{x}$$

$$\sigma_{\bar{x}} = \sqrt{\frac{(0.10-0.12)^2+(0.12-0.12)^2+(0.13-0.12)^2+(0.13-0.12)^2+(0.14-0.12)^2}{5\times(5-1)}}$$

= 0.01

$$x = \bar{X} \pm \sigma_{\bar{x}}$$

= **0.12± 0.01 mg**

For example, the MnO_2/graphene/Ni foam electrode prepared at 1 mA/cm^2, the mass of coated MnO_2 was also measured 5 times by ultra-precise balance. MnO_2 loaded mass was 0.07 mg, 0.08 mg, 0.09 mg, 0.10 mg, and 0.12 mg, respectively. The experimental data is calculated as follows:

$\bar{X}$= (0.07 + 0.08 + 0.09 + 0.10 + 0.12) ÷ 5

= 0.09

For a limited number of measurements, n= 5

$$\sigma_{\bar{x}} = \sqrt{\frac{\sum d_i^2}{n(n-1)}}$$

$$d = x - \bar{x}$$

$$\sigma_{\bar{x}} = \sqrt{\frac{(0.07-0.09)^2+(0.08-0.09)^2+(0.09-0.09)^2+(0.10-0.09)^2+(0.12-0.09)^2}{5\times(5-1)}}$$

= 0.01

$$x = \bar{X} \pm \sigma_{\bar{x}}$$

= **0.09± 0.01 mg**

Total mass loaded

$m_t = m_g + m_{MnO2}$

$= 0.12 + 0.09$

$= 0.21$

Error limits of m_t from statistical analysis

$= 0.01 + 0.01$

$= 0.2$

Hence, m_t = 0.21± 0.02 mg

Table S2. The specific capacitances (Cs, Cs′ and C_{sA}) of MnO_2/graphene/Ni foam prepared at different electrodeposition current densities obtained from CV curves under various scan rates.

Scan rate	Specific Capacitance											
	1 mA/cm²			5 mA/cm²			10 mA/cm²			15 mA/cm²		
	C_s (F/g)	C_s' (F/g)	C_{sA} (mF/cm²)	C_s (F/g)	C_s' (F/g)	C_{sA} (mF/cm²)	C_s (F/g)	C_s' (F/g)	C_{sA} (mF/cm²)	C_s (F/g)	C_s' (F/g)	C_{sA} (mF/cm²)
100 mV/s	202.9	179.3	36.5	155.8	138.5	38.4	76.7	89.1	27.6	52.4	67.2	26.2
80 mV/s	205.7	176.3	37.0	141.6	125.8	34.0	83.5	97.0	30.1	57.5	73.7	28.7
60 mV/s	214.3	183.7	38.6	170.0	151.1	40.8	91.4	106.1	32.9	63.5	81.4	31.7
40 mV/s	230.6	197.7	20.5	181.8	161.6	43.6	114.2	132.6	41.1	71.6	91.8	35.8
20 mV/s	261.6	224.2	47.1	202.0	179.6	48.5	122.9	142.7	44.2	86.3	110.6	43.1
10 mV/s	292.8	250.9	52.7	222.3	197.6	53.3	142.0	164.9	51.1	99.6	127.7	49.8

Notes:

(a) Cs: specific capacitance, calculated based on $C_s = \int \frac{|i|dV}{2m\,v\Delta V}$, m is the mass of deposited MnO_2.

(b) Cs′:specific capacitance, calculated based on $\boldsymbol{C_s'} = \int \frac{|i|dV}{M\,v\Delta V}$, and M is the mass of total loaded (graphene and MnO_2).

(c) C_{sA}': areal capacitances, $C_{SA}' = \int \frac{|i|dV}{S v\Delta V} = \frac{M}{S} \times \frac{1}{M} \int \frac{|i|dV}{v\Delta V} = C_S' \times \frac{M}{S}$

Table S3. The powder properties of MnO_2 and MnO_2/graphene materials.

Materials	Surface area (m².g⁻¹)	Pore volume (cm³. g⁻¹)	Pore size (nm)
MnO_2	158.5	0.3	3.7
MnO_2/graphene	179.2	0.3	7.8

Table S4. A comparison of the energy density and power density for the MnO_2//carbon system asymmetric supercapacitor in this work with the literature.

Materials	Electrolyte	Voltage Window (V)	Power Density (W/kg)	Energy Density (Wh/kg)	Reference
Zn-K-ATC/MnO_2//Zn-K-ATC	1 M Na_2SO_4	0–2.0	1000	46	42
MnO_2-GM-13-Ni//GM-13-Ni	1 M Na_2SO_4	0–2.0	500	125	43
GR/MWCNT/MnO_2// GR/MWCNT/MnO_2	0.5 M Na_2SO_4	0–1.8	426	35.5	44
N/P-HCS@MnO_2-30// N/P-HCS	1 M Na_2SO_4	0–1.8	449.8	32.2	45
GdMnO_2/$Ni(OH)_2$// PVA/KOH//Fe_3O_4/GO	PVA/KOH	0–1.6	2332	60	46
rGO/MnO_x//AC	[C_2MIm]BF_4	0–2.7	200	50	47
$NiCo_2S_4$/Co_9S_8//AC	1 M KOH	0–1.6	800	36.7	48
MnO_2/graphene//graphene	0.5 M Na_2SO_4	0–0.8	400	91	This work

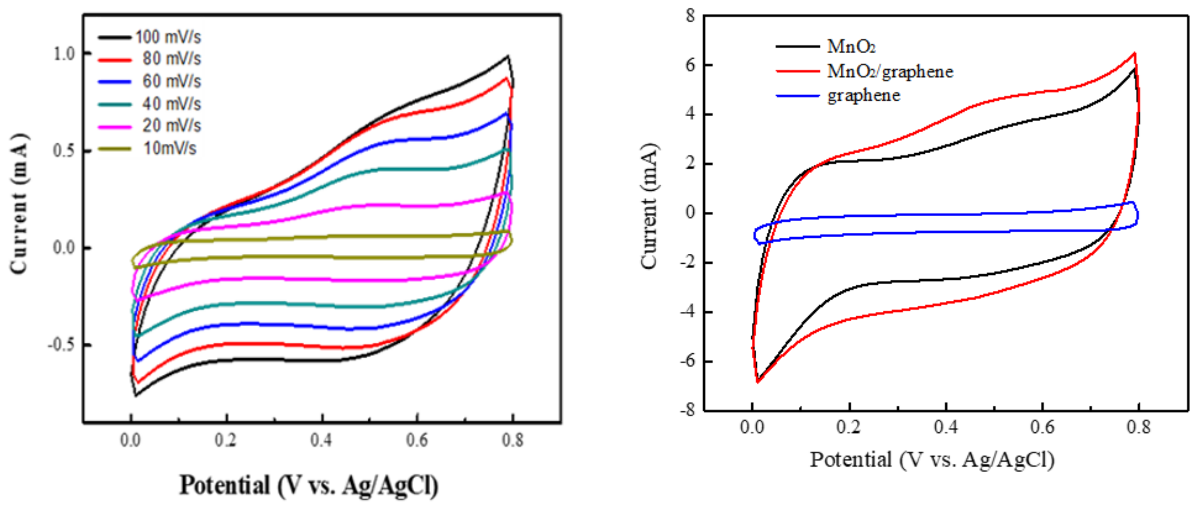

Figure S1. (**a**) CV curves of 0.12 mg loaded graphene/Ni at different scan rates; (**b**) CV curves of MnO2, MnO2/graphene/Ni and graphene/Ni (Ni: 54.35 mg, MnO2: 0.09 mg, and graphene: 0.12 mg).

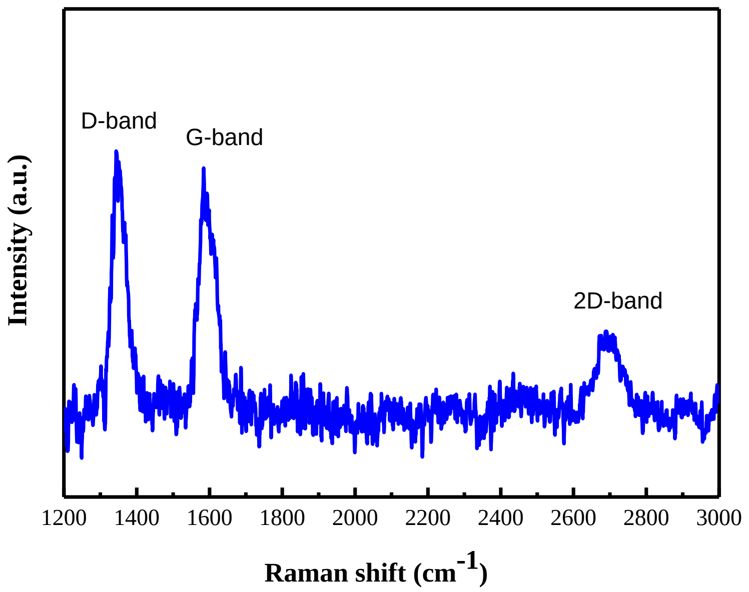

Figure S2. The Raman spectra of as–produced graphene.

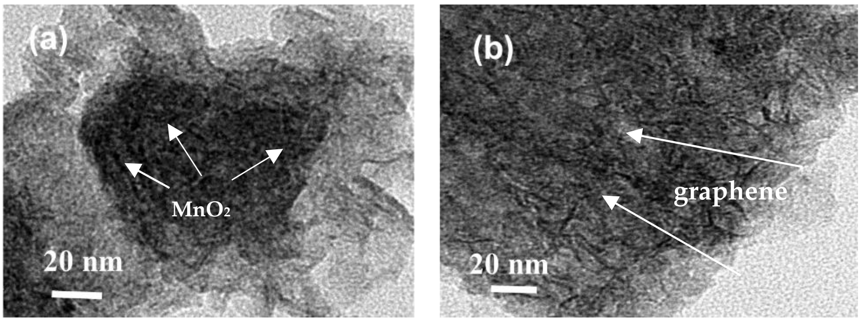

Figure S3. The TEM analysis for the as-obtained MaO_2 (**a**) and MnO_2/graphene material (**b**).

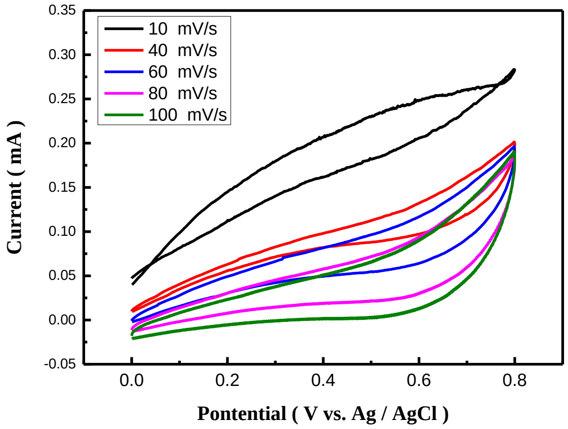

Figure S4. The CV characteristic curves of blank Ni foam at different scan rates.

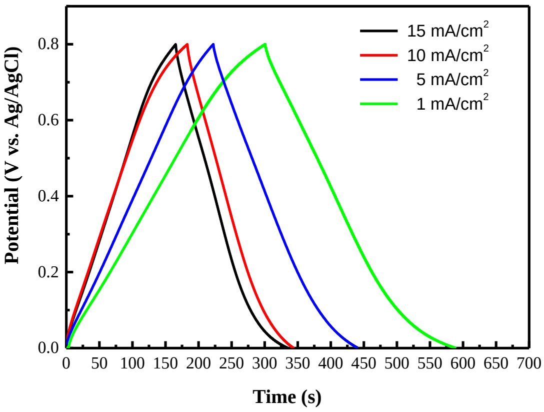

Figure S5. The GCD test for MnO_2/graphene/Ni electrodes obtained from different electrodeposition current densities examined at 1 A g^{-1}.

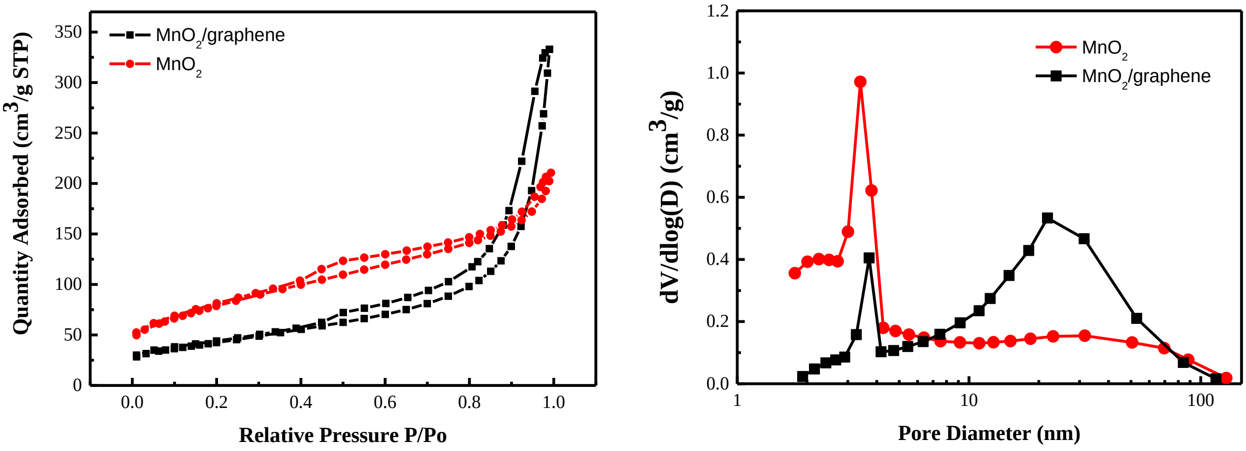

(a) (b)

Figure S6. BET specific surface area and pore size distribution analysis for MnO_2 and MnO_2/graphene materials. (**a**) N_2 isotherm adsorption-desorption analysis and (**b**) pore distribution analysis.
